# Clonally Related Plasmablastic Lymphoma Simultaneously Occurring with Diffuse Large B-Cell Lymphoma

**DOI:** 10.1155/2020/8876567

**Published:** 2020-12-01

**Authors:** Norisato Hashimoto, Tomoki Ueda, Shinichiro Hiraiwa, Takuma Tajiri, Naoya Nakamura, Kenji Yokoyama

**Affiliations:** ^1^Department of Hematology/Oncology, Tokai University Hachioji Hospital, 1838 Ishikawa-machi, Hachioji 1920032, Japan; ^2^Department of Pathology, Tokai University Hachioji Hospital, 1838 Ishikawa-machi, Hachioji 1920032, Japan; ^3^Department of Pathology, Tokai University, School of Medicine, 143 Shimokasuya, Isehara 2591193, Japan

## Abstract

Plasmablastic lymphoma (PBL) is a rare aggressive lymphoma. Although it was first described in HIV- (human immunodeficiency virus-) infected patients, PBL has been diagnosed in patients with other immunodeficiencies as well as in immunocompetent patients. PBL immunohistochemically expresses plasmacytic markers and lacks pan B-cell markers. The cells of origin of PBL are considered to be plasmablasts. MYC gene rearrangement and MYC overexpression are frequently found in PBL, but the pathogenesis of PBL is yet to be elucidated. Here, we report a case of composite lymphoma of PBL and diffuse large B-cell lymphoma (DLBCL); that is, PBL in the urinary bladder and DLBCL in the nasal cavity occurred simultaneously. We extracted DNA from the two lymphomas for polymerase chain reaction and sequenced the amplified immunoglobulin heavy variable genes and the complementarity-determining region- (CDR-) 3. The sequence of the CDR3 region of both tumors matched. MYC rearrangement was found in the bladder tumor but not in the nasal tumor. The patient was treated with R-CHOP (rituximab, cyclophosphamide, vincristine, doxorubicin, and prednisone), and durable remission had been obtained. The results of the DNA analysis indicated that both PBL and DLBCL emerged from common postgerminal B cells. This case may help to elucidate the pathogenesis of PBL.

## 1. Introduction

Plasmablastic lymphoma (PBL) is a rare aggressive lymphoma that was first described in 1997 as a new clinical entity related to human immunodeficiency virus infection and is recognized as a variant of diffuse large B-cell lymphoma (DLBCL) [1]. PBL has the morphologic and immunophenotypic features of plasmablasts that lack activating B-cell markers such as CD20 and harbor plasma cell markers such as CD138. Although PBL was initially reported as a neoplasm arising in the oral cavity of HIV-positive patients, PBL has been diagnosed in patients with other causes of immunodeficiency (e.g., transplant recipients), in immunocompetent patients, and in various anatomic locations. In the latest World Health Organization classification, PBL is categorized as a distinct mature B-cell lymphoma with specific pathologic and immunohistochemical characteristics [2].

Plasmablasts are considered to be the cells of origin of PBL, but the mechanisms of tumor evolution remain unclear [3]. Here, we report a case of composite lymphoma of PBL and DLBCL. We demonstrated that the two lymphomas derived from a common postgerminal center B cell. These findings provide insight into the possible tumor evolution of PBL from DLBCL.

## 2. Case Report

**Figure 1:**
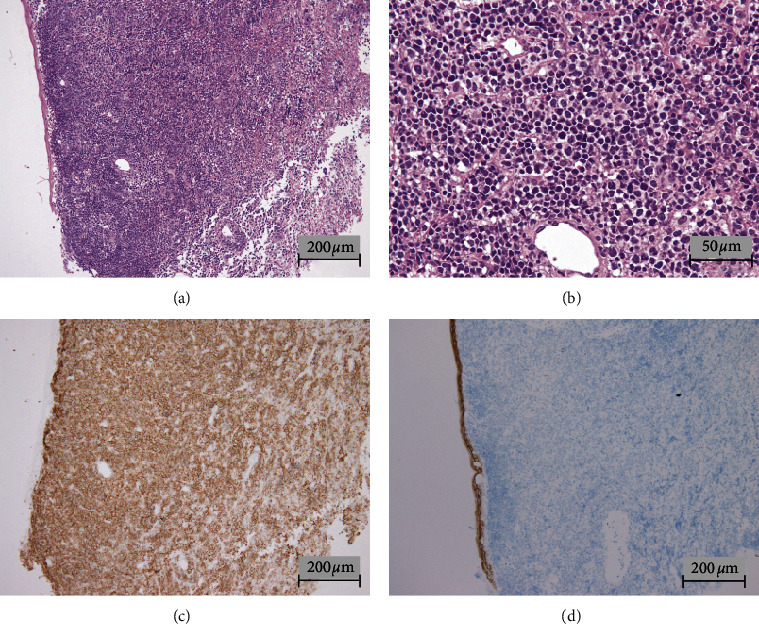
Hematoxylin and eosin- (H&E-) stained section of nasal mucosa (a, b). Immunohistochemical staining of CD20 (c) and CD138 (d). H&E staining showed diffuse proliferation of atypical lymphoid cells with prominent nucleoli and clear cytoplasm (a, b). Immunohistochemical staining revealed that atypical cells were positive for CD20 (c) and negative for CD138 (d).

A 37-year-old previously healthy man presented with swelling of the right nasal root and frequent urination. Otolaryngologic examination revealed thickened mucosa inside the right nasal cavity. Despite the use of both antibiotics and antihistamines, the symptoms deteriorated and the patient underwent diagnostic biopsy of the nasal mucosa. Hematoxylin and eosin (H&E) staining showed diffuse proliferation of atypical lymphoid cells with a prominent nucleolus and clear cytoplasm. Immunohistochemical staining revealed that the atypical cells were positive for CD20 and negative for CD138 (Figure 1). These cells were also positive for CD79a, PAX5, BCL2, BCL6, Mum-1, and CD45 and negative for CD56. A few cells were positive for in situ hybridization of Epstein–Barr virus- (EBV-) encoded RNA (data not shown). Based on these findings, the nasal tumor was diagnosed as nongerminal center B-cell (non-GCB) type DLBCL.

**Figure 2:**
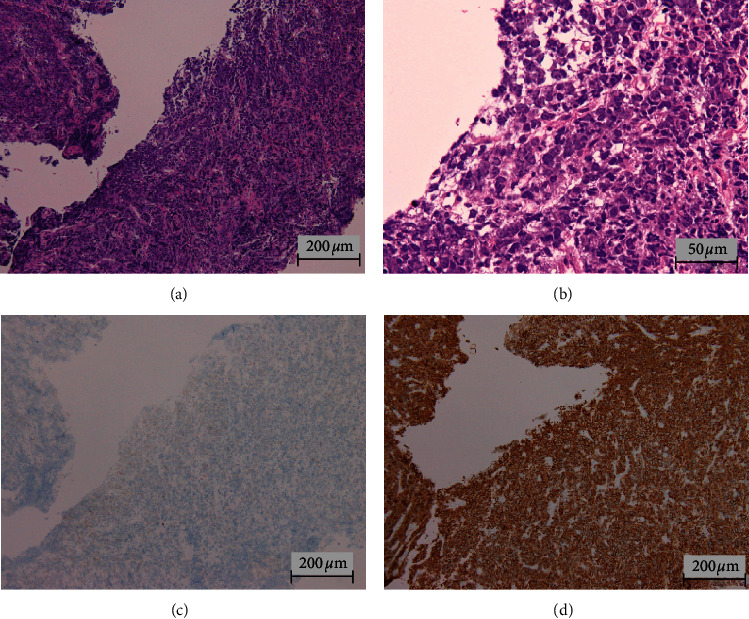
H&E-stained section of bladder tumor (a, b). Immunohistochemical staining of CD20 (c) and CD138 (d). H&E staining showed the proliferation of atypical lymphoid cells with abundant basophilic cytoplasm and pleomorphic nucleoli (a, b). Immunohistochemical staining revealed that these atypical cells were negative for CD20 (c) and positive for CD138 (d).

To evaluate the cause of frequent urination, ultrasonography of the urinary bladder was performed, and it revealed numerous irregular surfaced masses scattered on the wall of the urinary bladder. Biopsy specimens of one of the tumors showed the proliferation of atypical lymphoid cells with abundant basophilic cytoplasm and pleomorphic nucleoli. Immunohistochemical staining indicated that these atypical cells were negative for CD20 and positive for CD138 (Figure 2). These cells were also positive for CD56 and light chain kappa and negative for CD79a or PAX5 (data not shown). The serologic test for HIV was negative. According to these findings, the urinary bladder tumor was diagnosed as PBL. The patient did not complain of night sweats, weight loss, or fever. Blood laboratory tests indicated increased lactate dehydrogenase (334 U/ml), serum creatinine (1.67 mg/dl), and soluble interleukin-2 receptor (2650 U/ml) concentrations. Urinary tests revealed proteinuria and positivity for occult blood. Atypical cells were detected by urine cytology. A bone marrow trephine biopsy and cerebrospinal fluid cytology disclosed no lymphoma involvement. Fluorodeoxyglucose positron emission tomography/computed tomography (PET/CT) showed increased uptake at the lymph nodes on both the upper and lower sides of the diaphragm, and four extra nodal lesions in the right nasal mucosa, bilateral tonsils, prostate, and urinary bladder.

**Figure 3:**
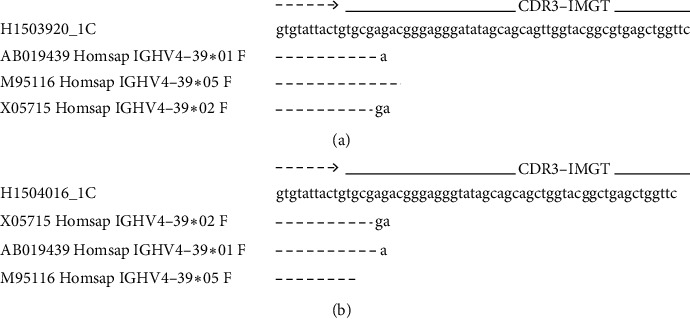
Sequences of the CDR3 region in the IGHV of the bladder tumor (a) and nasal tumor (b).The sequence of the CDR3 region in both tumors matched.

**Figure 4:**
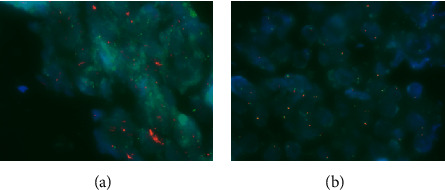
FISH analysis of the MYC of the bladder tumor (a) and nasal tumor (b). The split signal was detected in the bladder tumor, whereas no split signal was detected in the nasal tumor.

To identify the cell type of origin of the two lymphoma subtypes occurring simultaneously in one patient, we extracted DNA from frozen sections of each biopsy sample for polymerase chain reaction and sequenced the immunoglobulin heavy variable (IGHV) genes and the complementarity-determining region- (CDR-) 3, as previously described [4, 5] (see Supplementary Materials). The sequence of the CDR3 region of both tumors matched (Figure 3), indicating that both PBL and DLBCL emerged from a common clonal B cell. We also performed fluorescence in situ hybridization (FISH) in the biopsy specimens to detect MYC rearrangement. The split signal was detected in the bladder tumor, but not in the nasal tumor, indicating MYC rearrangement in the bladder PBL, but not in the nasal DLBCL (Figure 4).

This case was diagnosed as composite lymphoma of PBL and DLBCL and classified as stage 4A and IPI high/intermediate (elevated LDH, more than one extranodal lesion, and stage 4A). The patient underwent eight courses of R-CHOP (rituximab, cyclophosphamide, vincristine, doxorubicin, and prednisone) with four intrathecal infusions of methotrexate, cytarabine, and hydrocortisone to prevent central nervous system relapse, achieving a complete metabolic response based on PET/CT. Four years after completion of chemotherapy, he developed a soft tissue tumor on the cheek, and a biopsy specimen revealed recurrence of DLBCL.

## 3. Discussion

PBL is an aggressive lymphoma with the distinct morphologic features of large immunoblasts. The characteristic immunophenotype usually seen in PBL is the expression of plasmacytic markers, including CD138, CD38, and IRF4/MUM1, and the absence of pan B cell markers, including CD20 and PAX5. CD45 is weakly expressed or absent, and CD79a is often expressed in PBL [6]. The bladder tumor in our case was both morphologically and immunohistochemically compatible with PBL, and the nasal cavity tumor was morphologically and immunohistochemically compatible with non-GCB type DLBCL. Therefore, our case was diagnosed as composite lymphoma of PBL and DLBCL. Composite lymphoma is a rare manifestation of malignant lymphoma, which was first reported in 1954 [7], and there are several reports of non-Hodgkin lymphoma and Hodgkin lymphoma or two distinct subtypes of non-Hodgkin lymphoma. Most cases of composite lymphoma occur in the same organ, but some concurrently arise in different organs, such as in our case, the so-called discordant lymphoma [8].

The cells of origin of PBL are thought to be plasmablasts. Plasmablasts are precursor plasma cells derived from activated B cells. Several studies, including gene expression profiling studies, have investigated the pathogenesis of PBL, but it remains unclear. PBL was first reported in HIV patients, and EBV is detected in tumor cells of 74% of patients with HIV-associated PBL [9]. EBV, human herpes virus 8, MYC, p53, and BCL-6 gene aberrations may be involved in the development of AIDS-related lymphoma [10]. These mechanisms might also contribute to the pathogenesis of PBL, not only in HIV-infected patients but also in patients immunocompromised due to other causes as well as in immunocompetent individuals. Compared to HIV-associated PBL, approximately two-thirds of PBL cases in transplant recipients are EBV-positive and only half of the PBL cases in immunocompetent individuals are EBV-positive [11]. Chapman et al. [12] profiled the gene expression in 15 cases of PBL and compared them with the gene expression profiles of DLBCL and extramedullary (extraosseous) plasmacytoma. Overall, the gene expression profiles of PBLs were more similar to extramedullary (extraosseous) plasmacytoma than DLBCLs. They found that the transcriptional profile of PBL was distinct from that of DLBCL with regard to the activation of B-cell receptor signaling and the targets of MYC and MYB. They found that neither EBV positivity nor HIV status affected the gene expression profiles of PBL [12]. These findings suggest that the role of EBV or HIV in the pathogenesis of PBL may be limited.

MYC gene rearrangement is detected in up to 60% of PBL cases, and a common partner of the MYC gene is the immunoglobulin gene [13, 14]. Gene expression profiles of PBL show the overexpression of MYC at the mRNA level, which is correlated with MYC protein overexpression [12]. Montes-Moreno et al. [15] profiled the gene expression in 36 PBL cases and demonstrated that MYC overexpression is not restricted to MYC-translocated or MYC-amplified cases. These findings suggest that MYC overexpression has an important role in the pathogenesis of PBL. They also found recurrent somatic mutations in PRDM1 in 50% of PBL cases [15]. PRDM1 encodes the protein Blimp1, which is considered to regulate plasma cell differentiation. MYC rearrangement was found only in the bladder PBL in our case, suggesting that MYC rearrangement might be critical in the pathogenesis of PBL.

DNA sequence analysis in our case revealed that PBL in the urinary bladder and DLBCL in the nasal cavity had matched sequences at the CDR3 region of IGHV. These results indicated that both PBL and DLBCL were derived from the same clone. The clonal relation between PBL and low-grade lymphoma has been demonstrated in a case series [16]. Marini et al. [17] reported a case with transformed PBL from previously diagnosed DLBCL, but they did not show a clonal relation between the two tumors. To the best of our knowledge, this is the first case that demonstrates the simultaneous occurrence of clonally related PBL with DLBCL. Although gene expression profiling was not performed in our case, this case may help to elucidate the pathogenesis of PBL. We speculate that a master regulator of plasma cell differentiation, like BLIMP1, is aberrantly expressed in DLBCL, leading to the development of PBL.

R-CHOP is a standard treatment for patients with DLBCL, and two-thirds of nonelderly patients with advanced-stage DLBCL can be cured with 6 to 8 cycles of R-CHOP. A standard treatment for PBL has not been established due to the rarity of the disease and its aggressive clinical course. Median survival time for PBL patients is estimated to be less than 1 year. A standard-dose CHOP-like regimen is considered inadequate because it provides the least possibility of cure [3]. The National Comprehensive Cancer Network recommends more intensive chemotherapy [18]. Bortezomib, a proteasome inhibitor that is a highly effective drug in multiple myeloma, is a promising agent for PBL because it targets plasma cells [19]. A recent study reported that the addition of bortezomib to dose-adjusted EPOCH (etoposide, prednisolone, vincristine, cyclophosphamide, hydroxydaunorubicin) led to a better response and survival in both HIV-positive and HIV-negative PBL patients, but the study was limited due to its retrospective design and small sample size [20]. Our case had composite lymphoma of PBL and DLBCL, and we selected R-CHOP as the first-line therapy. Our patient achieved a complete metabolic response after completing eight courses of R-CHOP, and durable remission for four years had been obtained. PBL does not usually express CD20, and therefore, the addition of rituximab to the chemotherapy may not improve the response to chemotherapy. In the largest retrospective cohort study of PBL, CD20 was positive in 10% of patients, while 18% of patients received rituximab combined with or without chemotherapy [21]. When combined with chemotherapy, rituximab tended to improve the complete remission (CR) rate compared to chemotherapy alone, irrespective of CD20 or EBV positivity [21]. Considering our case and this cohort study, the addition of rituximab to the chemotherapy might be beneficial in selected cases of PBL. Our patient had been in CR for four years, but DLBCL relapsed as a soft tissue tumor. Serum soluble interleukin-2 receptor was markedly elevated at diagnosis, and increased serum soluble interleukin-2 receptor at diagnosis has been reported to be related to poor prognosis of DLBCL [22, 23]. Autologous stem cell transplantation (ASCT) in first CR has been shown to improve the outcomes of PBL patients [24]. Although the optimal treatment of composite lymphomas has not been established, ASCT after completion of R-CHOP might reduce the risk of recurrence of lymphoma in our case.

## Data Availability

All data generated or analyzed during this study are included within this article and its supplementary information files.

## Supplementary Materials

Supplementary Materials The primer sequences were used to amplify the immunoglobulin heavy chain gene by seminested polymerase chain reaction (PCR) as described previously [13, 14]. The PCR products were treated with EXO SAP IT (USB, Affymetrix Japan) according to the supplier's protocol and sequenced using the ABI PRISM 3100 Genetic Analyzer (Applied Biosystems Japan). Sequences were compared with the germline sequences using the IMGT site
