# Supplementary material for: Clonally Related Plasmablastic Lymphoma Simultaneously Occurring with Diffuse Large B-Cell Lymphoma
Source: Case Rep Hematol. 2020 Dec 1;2020:8876567. doi: 10.1155/2020/8876567 (PMC7723488; doi:10.1155/2020/8876567)
Supplement: Supplementary Materials — The primer sequences were used to amplify the immunoglobulin heavy chain gene by seminested polymerase chain reaction (PCR) as described previously [13, 14]. The PCR products were treated with EXO SAP IT (USB, Affymetrix Japan) according to the supplier's protocol and sequenced using the ABI PRISM 3100 Genetic Analyzer (Applied Biosystems Japan). Sequences were compared with the germline sequences using the IMGT site [file 8876567.f1.docx]

5'-CCGGRAARRGTCTGGAGTGG -3', as the upstream consensus V region primer (FR2A)

5'-CTTACCTGAGGAGACGGTGACC -3', as the consensus J region primer (LJH)

5'-GTGACCAGGGTNCTTGGCCCC-3', as a consensus J region primer (VLJH)
